# Evaluation of procoagulant imbalance in Cushing’s syndrome after short- and long-term remission of disease

**DOI:** 10.1007/s40618-021-01605-5

**Published:** 2021-06-11

**Authors:** E. Ferrante, A. L. Serban, M. Clerici, R. Indirli, E. Scalambrino, G. Carosi, L. Padovan, M. Locatelli, M. Arosio, F. Peyvandi, G. Mantovani, A. Tripodi

**Affiliations:** 1 Endocrinology Unit, Fondazione IRCCS Ca’ Granda Ospedale Maggiore Policlinico di Milano, Via Francesco Sforza, 35, 20143 Milano, Italy; 2 Department of Experimental Medicine, Sapienza University of Rome, Rome, Italy; 3 Angelo Bianchi Bonomi Hemophilia and Thrombosis Center, Fondazione IRCCS Ca’ Granda Ospedale Maggiore, Milano, Italy; 4 Department of Clinical Sciences and Community Health, University of Milan, Milano, Italy; 5 Neurosurgery Department, Fondazione IRCCS Ca’ Granda Ospedale Maggiore Policlinico di Milano, Milano, Italy; 6 Department of Pathophysiology and Transplantation, University of Milan, Milan, Italy

**Keywords:** Cushing’s syndrome, Hypercoagulability, Thrombin generation assay, Disease remission

## Abstract

**Objective:**

Patients with Cushing’s syndrome (CS) are at high risk of venous thromboembolism related to a hypercoagulability due to procoagulant imbalance. However, whether these alterations are reversible after disease remission is still unclear. The endogenous thrombin potential (ETP) measured with and without the addition of thrombomodulin provides a global representation of coagulation and previous data confirmed hypercoagulable profile in patients with active hypercortisolism. Aim of this study was to assess the short- and long-term modification of ETP in patients with CS after disease remission.

**Design and methods:**

Nineteen patients with CS for whom surgical remission was achieved, were prospectively evaluated for clinical characteristics, cortisol secretion profile and ETP at different time points: (i) before surgical intervention; (ii) after 6 months and (iii) 5 years from the time of persistent remission. Nineteen healthy subjects matched for age and gender were also evaluated as control group.

**Results:**

Before surgery, patients showed higher ETP-ratio (with/without thrombomodulin) than controls (0.62 ± 0.09-vs-0.56 ± 0.09, *p *= 0.034). No significant correlation between ETP-ratio and cortisol secretion was found. 6 months after remission, ETP-ratio was still significantly increased compared to controls (0.64 ± 0.09-vs-0.56 ± 0.09, *p *= 0.01), but was similar to baseline (0.64 ± 0.09-vs-0.62 ± 0.09, *p *= 0.87). At 5 years, ETP-ratio showed a significant decrease (0.55 ± 0.14-vs-0.62 ± 0.09, *p *= 0.02) and was comparable to controls (0.55 ± 0.14-vs-0.56 ± 0.09, *p *= 0.7).

**Conclusions:**

Plasma hypercoagulability detected in patients with active hypercortisolism persists at short-term evaluation and seems to be completely reversible after long-term remission of disease. These data, as part of a whole evaluation of thrombotic risk, can contribute to make appropriate therapeutic choice in these patients.

## Introduction

Cushing syndrome (CS) is associated with a high venous thromboembolic (VTE) risk with an odds ratio of 17.8 (95% CI 15.24–20.85) when compared to general population [1]. The incidence of VTE events increases during the first months after surgery and then progressively decreases throughout follow-up, although it is likely to remain higher than in general population even in case of surgery [2, 3].

As known, thrombosis is related to plasma hypercoagulability that was extensively investigated also in CS. In particular, in patients with active hypercortisolism, previous studies showed increased levels of various procoagulant factors involved in the intrinsic and common pathways, mainly von Willebrand and factor VIII[ 1, 4–12] but also IX, X and XI [4, 9, 13]. However, an increase of anticoagulant factors such as protein C, protein S and antithrombin [1, 9, 11, 14, 15], as well as fibrinolytic markers was also observed [7, 9, 13]. Therefore, although the increase of anticoagulant factors is usually interpreted as a compensatory response [16], measurement of single coagulation factors does not allow to assess the whole haemostatic profile in CS patients. Measurement of the activated partial thromboplastin time (aPTT), a global test that assesses both intrinsic and common pathway of coagulation could be alternatively used. aPTT is shortened in CS when compared to controls [1, 4, 6, 8–10, 12, 14, 17, 18], and is a likely independent risk factor for VTE [19]. However, aPTT is not fully representative of coagulation in vivo as plasma in this test starts to clot when less than 5% of thrombin is formed [20].

In 2008, Hemker and collaborators developed a relatively complex procedure that assesses plasma thrombin generation and decay, called thrombin generation assay (TGA) [21]. TGA provides a global account of the coagulation profile (i.e. the balance between pro- and anticoagulant factors) as presumably occurs in vivo. The main parameters of thrombin generation curve or thrombogram are: the lag time—defined as the time (minutes) elapsing from coagulation activation to the initiation of thrombin generation; the thrombin peak (nM), the time to reach the peak (minutes); the area under curve also called endogenous thrombin potential (ETP) (nM x minutes), and the ETP-ratio (ETP with/without thrombomodulin). Hypercoagulability is characterized by a short lag time, high peak, short time to peak, high ETP and ETP-ratio [22].

In CS, Koutroumpi and collaborators observed that patients with active disease presented significantly shortened lag time, higher thrombin peak and ETP when compared to nondiabetic nonobese controls, but similar to patients with metabolic syndrome, except for lag time which was significantly shorter in the latter group [11]. A more recent study by our group compared TGA in CS patients to age and gender-matched controls and concluded that all TGA parameters were modified towards hypercoagulability and were associated with increased levels of neutrophil extracellular trap (NET)-related factors [15]. These previous publications assessed the coagulation profile through TGA in patients with active hypercortisolism but, to our knowledge, there are no studies evaluating TGA in CS during follow-up after surgical remission, when coagulation profile and VTE risk are expected to change.

The aim of the present study was to investigate the short- and long-term modifications of TGA in patients with CS after surgical intervention and to compare them to a control group matched for gender and age.

## Patients and methods

### Patients

Between 2011 and 2014, 31 patients were diagnosed with CS at our Endocrinology Unit. 12 out of 31 were excluded from the study for the following reasons: anticoagulant therapy (*n *= 1), refusal of surgical treatment (*n *= 2), persistent hypercortisolism after surgical intervention (*n *= 4), recurrence of CS (*n *= 2) and premature follow-up discontinuation (*n *= 3). The final cohort includes 19 patients with active hypercortisolism (16 patients affected by CD, two with adrenal CS and one with ectopic CS). The female to male ratio was 12/7 and the mean age 44.9 ± 11.3 years.

Diagnosis of hypercortisolism was based on clinical features and standard biochemical evaluation including urinary free cortisol (UFC) levels and low-dose dexamethasone suppression test (LDDST) [23]. To establish the origin of hypercortisolism, the measurement of ACTH, CRH test, HDDST, pituitary MRI and abdomen CT were performed as appropriate [24].

All patients underwent surgical treatment: transsphenoidal surgery (TSS) for CD, unilateral adrenalectomy for adrenal CS and bilateral adrenalectomy for ectopic CS.

Clinical characteristics, cortisol secretion profile and TGA parameters were collected before surgical intervention, at 6 months (median, range 3–8; short-term follow-up) and 5 years (median, range 4–6; long-term follow-up) after surgery. Moreover, TGA was also evaluated in a subgroup of eight patients after 15 months (median, range 11–17) from surgery.

Body mass index (BMI), systolic and diastolic blood pressure (SBP and DBP), fasting blood glucose and glycated hemoglobin were also evaluated. Overweight and obesity was defined as BMI between 25 and 29.9 and BMI > 30 kg/m2, respectively; hypertension was defined as SBP of 140 mm Hg or higher and/or DBP of 90 mm Hg or higher and/or current medical treatment; prediabetes was defined as fasting glucose between 100 and 125 mg/dl and/or glycated hemoglobin between 5.7 and 6.4%. Finally, diabetes mellitus was defined as fasting glucose ≥ 126 mg/dl and/or glycated hemoglobin ≥ 6.5% and/or current use of antidiabetic drugs or insulin.

Biochemical remission of disease was maintained during the entire follow-up period in all patients. The diagnostic criteria of remission were adrenal insufficiency, or normal pituitary/adrenal axis function in the presence of normal UFC levels, normal value of late-night salivary cortisol and positive response to LDDST. In case of primary or secondary adrenal insufficiency, as far as other pituitary deficits, adequate substitutive therapy was prescribed.

Nineteen outpatients matched for gender, age and metabolic features, without clinical signs of hypercortisolism and personal or family history of thrombosis/hemorrhage and free from drugs known to affect coagulation, were evaluated as control group.

### Methods

#### Blood collection and plasma preparation

Venous blood samples were collected into vacuum tubes containing 109 mM trisodium citrate at a proportion of 1:10 anticoagulant:blood. Blood was centrifuged within 30 min at 3000 g for 20 min (controlled room temperature) and supernatant plasma was immediately frozen in liquid nitrogen and stored at − 70 °C until testing.

#### Thrombin generation assay

Thrombin generation was assessed according to Hemker et al. [21] as described [25] and based on the activation of coagulation after addition to plasma of human recombinant tissue factor (1 pM) (Recombiplastin 2G, Werfen, Orangeburg, NY) and synthetic phospholipids (1.0 μM) (Avanti Polar, Alabaster, Alabama) as coagulation triggers. Testing was carried out in two plasma aliquots, with and without addition of rabbit thrombomodulin (TM) (Haematologic Technologies) (6 nM). Registration of thrombin generation was obtained with a fluorogenic substrate (Z-Gly-Gly-Arg-AMC HCl, Bachem, Bubendorf, Switzerland) (617 μM) by means of a fluorometer (Fluoroskan Ascent^®^, ThermoLabsystem, Helsinki, Finland). The readings were recorded and analyzed with a dedicated software (Thrombinoscope^TM^, Thrombinoscope BV, Maastricht, The Netherlands), which displays the curve of thrombin concentration as a function of time (*thrombogram*) and calculates the lag time; the thrombin peak; the time to reach the peak; the area under the curve, defined as endogenous thrombin potential (ETP); the velocity index (nM/min), defined as $\left[ {{\text{Peak}}/\left( {{\text{time}}\;{\text{to}}\;{\text{peak}}\; - \;{\text{LagTime}}} \right)} \right]$. Results were also expressed as ETP-ratio, i.e. the ratio of ETP measured in the presence of TM (ETP-TM^+^) to the ETP measured in its absence (ETP-TM^−^). To limit analytical variability, an equal number of samples from patients and controls were tested in the same run.

### Statistical analysis

Data were expressed as mean ± standard deviation for normally distributed continuous variables, median and interquartile range (IQR) or range for non-Gaussian data and proportion for categorical parameters. The latter were analysed using the *χ*^2^ test or the Fisher exact test if the expected value was < 5. Continuous parameters with normal distribution were compared using the *t* test and non-Gaussian data using the non-parametric test of Mann–Whitney. We assessed the relationship between variables through linear regression or Spearman’s rank correlation test as appropriate. Two-sided *p* value was considered statistically significant when < 0.05. All statistical analyses were performed using SPSS, version 26 (IBM, Chicago, IL).

ETP-ratio was considered as the outcome variable for sample size calculation. On the basis of previous published data in CS patients [15], postulating a mean variation of matched pairs of 20% in ETP-ratio as the effect, evaluation of at least 9 pairs of subject has been estimated to be necessary to reject the null hypothesis that this difference is zero with a power of 90% and a Type I error probability of 5%.

## Results

### Baseline (CS before surgery and controls)

Demographic, biochemical and metabolic characteristics of patients and controls are summarized in Table 1.

**Table 1:** General characteristics of patients and controls

General characteristics	Controls (19)	Patients (baseline, 19)		Patients (6 months, 19)	Patients (5 years, 19)
Females (*n*, %)	12 (63.1%)	12 (63,1%)		/	/
Age at diagnosis (years, mean ± SD)	44,4 ± 10,8	44,9 ± 11,3		/	**/**
BMI (Kg/m^2^, mean ± SD)	26.1 ± 4.0	26.0 ± 5,8		24.7 ± 3.7*	24.8 ± 4.9*
Basal serum cortisol [µg/dl, median (IQR)]	/	20,3 (17–29)		/	/
Cortisol post DXM 1 mg [µg/dl, median (IQR)]	/	12,7 (4.2–17)		/	/
UFC (xULN)	/	1,65 (0.9–2.6)		/	/
Overweight or obesity (*n*)	10/19	9/19		7/19	8/19
Hypertension (*n*)	8/19	9/19		7/19	7/19
Dysglycemia (*n*/total)	5/19	5/19		1/19	1/19
Prediabetes	3/19	2/19		0/19	0/19
Diabetes mellitus	2/19	3/19		1/19	1/19
Hypoadrenalism (*n*/total)	/	/		19/19	13/19

*SD* standard deviation, *IQR* interquartile range

*p < 0.05 vs patients at baseline

There was no significant difference in distribution of gender, age and metabolic comorbidities between patients and controls (Table 1).

**Fig. 1:**
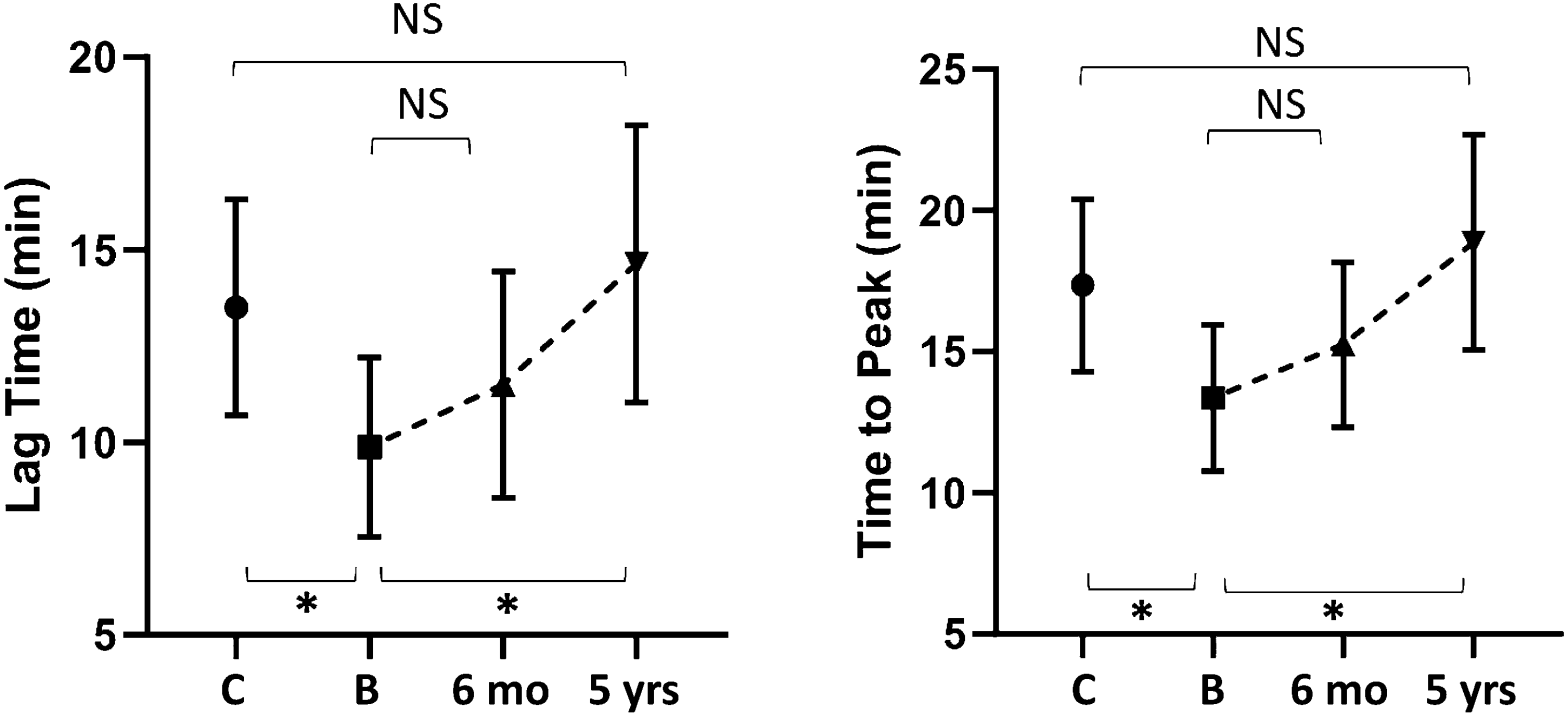
Lag time and time to peak in patients and controls, represented as mean and standard deviation. C: controls; B: CS at baseline, 6 months: CS 6 months after surgery; 5 years: CS 5 years after surgery. *Indicates statistically significant difference (*p* value < 0.05)

**Fig. 2:**
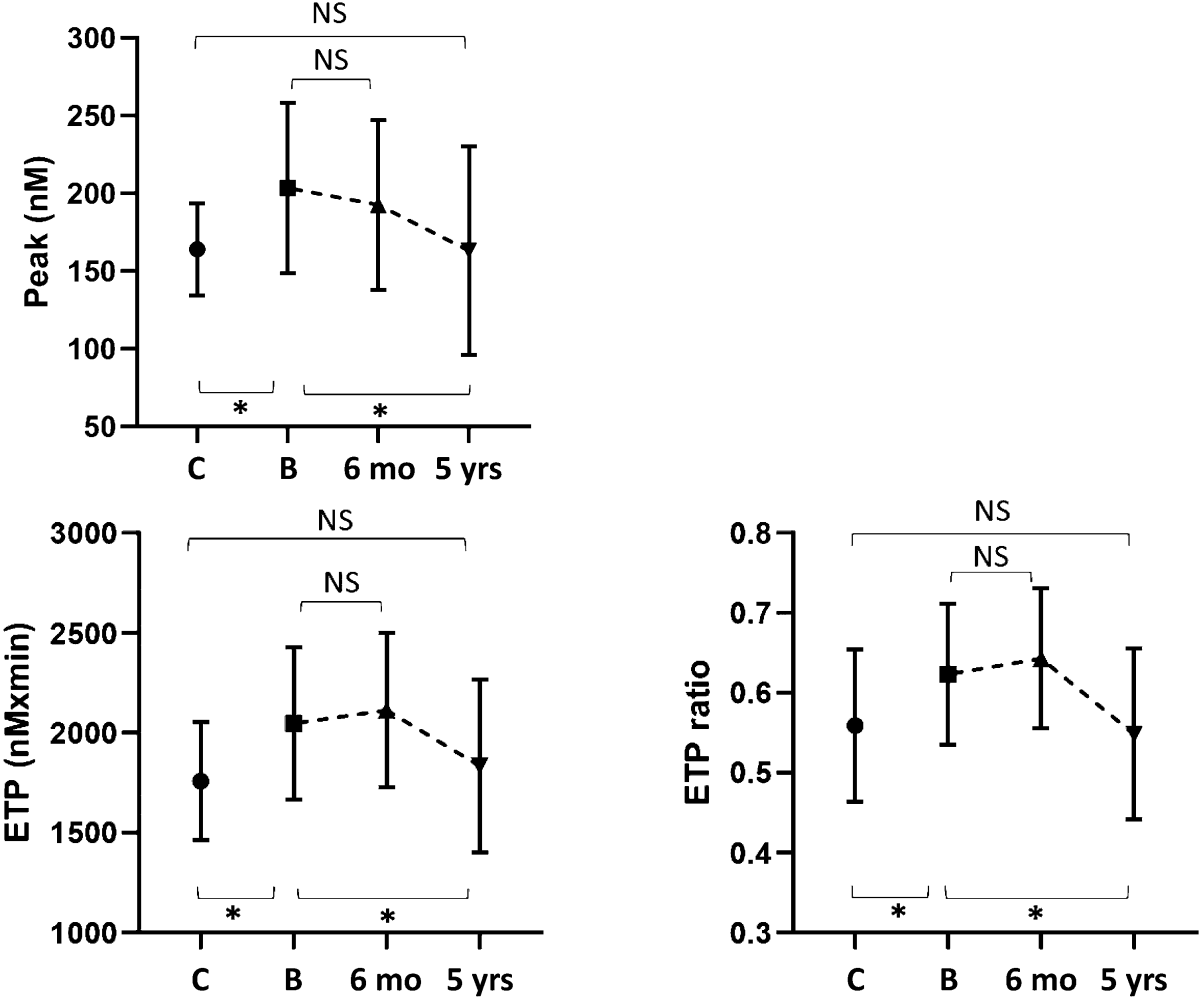
Peak, endogenous potential of thrombin (ETP, without TM) and ETP ratio in patients and controls, represented as mean and standard deviation. C: controls; B: CS at baseline, 6 months: CS 6 months after surgery; 5 years: CS 5 years after surgery. *Indicates statistically significant difference (*p* value < 0.05)

At baseline, lag time and time to peak were shorter in patients than controls (*p *< 0.001); ETP and peak were higher in patients than controls (< 0.05); ETP-ratio (with/without thrombomodulin) was higher in patients than controls (< 0.05) (Table 2, Figs. 1 and 2). The above modifications indicate a hypercoagulable profile in CS patients.

**Table 2:** TGA parameters in patients with CS before and after surgical cure and controls

	Controls	CS at baseline	CS after 6 months	CS after 5 years	CS baseline vs controls	CS baseline vs 6 months	CS 6 months vs controls	CS baseline vs 5 years	CS 5 years vs controls
Lag time (min)	13.5 ± 2.8	9.8 ± 2.3	11.5 ± 3	14.6 ± 3.6	< 0.001	0.141	0.047	< 0.001	0.28
Peak (nmol/L)	163 ± 29	203 ± 54	192.5 ± 54	163 ± 67	0.025	0.364	0.053	0.007	0.96
Time to peak (min)	17.35 ± 3	13.5 ± 2.6	15.2 ± 3	18.8	< 0.001	0.092	0.001	< 0.001	0.18
ETP (nmol/L × min)	1756 ± 295	2045 ± 381	2113 ± 385	1835 ± 433	0.013	0.266	< 0.001	0.022	0.52
ETP-ratio	0.56 ± 0.09	0.62 ± 0.09	0.64 ± 0.08	0.55 ± 0.1	0.039	0.876	0.01	0.02	0.74

Data are expressed as mean ± standard deviation. A *p* value < 0.05 was considered statistically significant

*ETP* endogenous thrombin potential. ETP-ratio, ETP (with/without thrombomodulin)

In CS patients, no significant correlation between TGA parameters and disease duration, basal cortisol levels, cortisol levels after 1 mg dexamethasone suppression test and UFC values were found. Likewise, BMI, hypertension and prediabetes/diabetes mellitus were not associated with the coagulation profile in both groups.

### Hypopituitarism after TSS in CD patients

After pituitary surgery, hypoadrenalism was recorded in all patients. A complete evaluation of pituitary function was then performed 6–8 weeks after surgery. In ten out of 16 patients (62.5%), at least one additional pituitary deficit besides hypoadrenalism was present. In detail, central hypothyroidism, central hypogonadism and diabetes insipidus were diagnosed in 5, 3 and 3 patients, respectively. In all subjects, replacement therapy was prescribed at diagnosis. Furthermore, 8 patients presented growth hormone deficiency, but the replacement therapy was postponed to 12 month follow-up to ascertain persistent disease remission. Two patients had panhypopituitarism.

### Short-term remission (6 months after surgery)

6 months after surgical treatment, all subjects still had adrenal insufficiency and were receiving substitutive therapy with physiologic doses of cortisone acetate (25 mg/day divided in two or three doses). Lag time and time to peak increased when compared to baseline, but did not reach the levels of controls (Fig. 1 and Table 2); peak decreased when compared to baseline, but did not reach the levels of controls (Fig. 2 and Table 2); ETP and ETP-ratio showed a slight increase (Fig. 2, Table 2). Altogether, these results indicate that hypercoagulability is persistent after short-term remission of hypercortisolism.

At this time point, untreated GH deficiency did not influence coagulation parameters. Finally, evaluation of TGA in a subgroup of 8 patients 15 months after surgery did not reveal significant difference in respect to data collected at 6 months (data not shown).

### Long-term remission (5 years after surgery)

At long-term follow-up [median 5 (range 4–6 years)], recovery of adrenal function was observed in 6 patients affected by CD (6/16, 37.5%). As stated above, normal UFC levels as well adequate cortisol suppression after LDDST confirmed disease remission. The remaining subjects were still on replacement therapy.

Nonparametric analysis did not show any significant difference in lag time, peak, time to peak, ETP and ETP-ratio in patients with recovery of adrenal function compared to those receiving cortisone acetate (data not shown).

Overall, CS patients showed a significant increase of lag time and time to peak as well as a significant decrease of peak, ETP and ETP-ratio when compared to baseline and were not significantly different from those observed for controls (Table 2, Figs. 1 and 2). Therefore, these parameters show that sustained remission of disease allows a substantial correction of the baseline procoagulant imbalance.

No VTE events were recorded in CS patients.

## Discussion

Chronic hypercortisolism determines dramatic biochemical and clinical alterations leading to several comorbidities and to a significantly higher mortality risk than those observed in the general population [26, 27]. In a previous study, we showed that CD patients at baseline possess a state of hypercoagulability as shown by increased thrombin generation. Whether and to what extent the baseline hypercoagulability of CD patients is reversible after disease remission is still incompletely understood. A previous paper showed that after 12 months from surgery, normalization of some coagulation parameters was achieved only in remitted patients, without any improvement in those with persistent disease [28]. Furthermore, Kastelan et al. reported a significant decrease of most pro- and anticoagulants 6 months after surgical cure, thus suggesting an apparent coagulation asset which was comparable to that of controls [29]. However, the above studies measured only single components of coagulation (either pro- or anticoagulant factors) and none used thrombin generation procedures to assess for global coagulation. In this study, we sought to fill this gap by investigating patients with CS at different time points after surgery by means of thrombin generation procedures carried out in the presence or absence of thrombomodulin.

Our results show that the procoagulant imbalance observed at baseline is still persistent after 6 months from surgery and is completely reversed after long-term remission, when all the TGA parameters in patients appear comparable to those of the control group.

The short-term persistence (after 6 months from surgery) is in line with observations made in other studies that the risk of VTE (the possible clinical outcome of hypercoagulability), is still elevated in a period between 3 to 12 months in CS, despite surgical cure and draws attention to the complex and not fully explained link between hypercortisolism and procoagulant imbalance [3].

Notably, different studies reported a variable and inconsistent association between basal cortisol levels, UFC, late-night salivary cortisol and coagulation factors [4, 8, 9, 14, 16]. In addition, regression analysis performed by Wagner et al. did not find any association between single factors and UFC. Regarding TGA, Koutroumpi et al. described a positive correlation between lag time and night salivary cortisol [11], while in our previous study we found a significant correlation between ETP-ratio and both basal cortisol levels and UFC [15]. On the other hand, in the present study, we failed to demonstrate any association between TGA parameters and basal cortisol, or cortisol after 1 mg dexamethasone suppression test and UFC values, although the analysis of the association could have been affected by the relatively small number of patients.

In CS patients, as in general population, other comorbidities can nonetheless contribute to increase thromboembolic risk. In our cohort, one patient was affected by ectopic CS, which may present a different coagulation pattern as well VTE risk related to the underlying neoplasm. However, since the majority of studies did not distinguish between the different aetiologies of hypercortisolism, definitive data on this issue are still lacking [1]. Moreover, the procoagulant imbalance detected by TGA has been shown to be associated with obesity, hypertension and diabetes [11, 30–32]. In addition, obesity and diabetes represent independent risk factors of VTE [33]. However, in the present study, the control group had the same prevalence of metabolic disorders (i.e. BMI, prediabetes/diabetes mellitus, hypertension) as CS patients and TGA parameters did not change at short-time follow-up despite a significant decrease of BMI. Moreover, coagulation parameters were not associated with metabolic features both in CS patients at baseline and in control group. Along with previous description of a persistent coagulation imbalance in not-cured patients as well as the absence of VTE events in patients surgically treated for non-functioning pituitary adenomas [2, 28], these data support the central role of cortisol in the context of coagulation. Importantly, at long-term follow-up we did not find any significant difference in TGA parameters between patients on substitutive therapy and those with recovery of adrenal function. Of note, the proportion of patients with adrenal insufficiency at long-term follow-up was quite high, but this may be due to the strict inclusion criteria we applied as well as the small cohort of subjects evaluated. Considering also that 6 months after surgery, all patients were receiving physiologic doses of cortisone acetate, we can speculate that replacement therapy do not significantly alter coagulation profile in these subjects.

Furthermore, it is well known that VTE risk is particularly high in the early postoperative period. After surgical treatment, a paradoxical increase of coagulation factors (in particular factor VIII) related to the lack of anti-inflammatory effect of hypercortisolism has been postulated and may contribute to explain the delayed normalization observed in our patients [1, 14].

In view of the high postoperative risk of VTE events, different interventions such as early mobilization, elastic stockings and prolonged thromboprophylaxis have been recommended [10, 14]. Since our data show that the hypercoagulability is persistent for at least 6 months postoperatively, the duration of the antithrombotic prophylaxis should be tailored accordingly and also taking into consideration other risk factors of VTE and bleeding. Recently, a risk assessment model for CS patients, including age (≥ 69 years), reduced mobility, acute severe infections, previous cardiovascular events, midnight plasma cortisol level (> 3.15 times the upper limit of the reference range) as well as shortened aPTT as independent risk factor for VTE, has been proposed and could represent a useful tool for this purpose [19].

Our study has some limitations. First, the relatively small number of patients limits the opportunity to clarify the role of cortisol and metabolic comorbidities in determining hypercoagulability in CS patients. However, it should be recognized that follow-up studies in this category of patients are inherently difficult. Furthermore, this is the first study that so far attempted the post-operative evaluation of CS patients with respect to hypercoagulability assessed by a last generation global coagulation procedure mimicking what presumably occurs in vivo. Second, in this study we were unable to measure prospectively other non-coagulation parameters such as NET-related molecules, for which we recently demonstrated a role in determining the procoagulant imbalance in the active phase of the disease [15]. The extent of variation of these factors as well as circulating microparticles and markers of endothelial dysfunction, could be relevant to the thromboembolic risk. Third, given the long period between short- and long-term evaluations, our study cannot indicate when normalization of hypercoagulability exactly occurs. Additional data on a subgroup of eight patients evaluated at 15 months after disease remission suggest that TGA do not normalize even at this time point, however, the paucity of cases precludes further consideration.

In conclusion, endogenous hypercortisolism determines a hypercoagulable state that persists 6 months after surgery but is completely reversed in the long-term follow-up in patients who achieve disease remission. Although understanding the exact nature of the procoagulant imbalance observed in CD patients requires further studies, our data should be taken into consideration when evaluating thrombotic risk and choice/duration of antithrombotic prophylaxis in this category of patients.
